# Do Best-Selected Strains Perform Table Olive Fermentation Better than Undefined Biodiverse Starters? A Comparative Study

**DOI:** 10.3390/foods9020135

**Published:** 2020-01-28

**Authors:** Antonio Paba, Luigi Chessa, Elisabetta Daga, Marco Campus, Monica Bulla, Alberto Angioni, Piergiorgio Sedda, Roberta Comunian

**Affiliations:** 1 AGRIS Sardegna, Agenzia regionale per la ricerca in agricoltura, Loc. Bonassai, km 18.600 S.S. 291, 07100 Sassari, Italy; apaba@agrisricerca.it (A.P.); lchessa@agrisricerca.it (L.C.); edaga@agrisricerca.it (E.D.); campus@portocontericerche.it (M.C.); monica.bulla@libero.it (M.B.); psedda@agrisricerca.it (P.S.); 2 Current affiliation: Porto Conte Ricerche S.r.l, S.P. 55 Porto Conte-Capo Caccia km 8,400 Loc. Tramariglio, 07041 Alghero (SS), Italy; 3 Dipartimento di Scienze della Vita e dell’Ambiente, Università di Cagliari, Viale S. Ignazio da Laconi, 13, 09123 Cagliari, Italy; aangioni@unica.it

**Keywords:** undefined biodiverse starters, autochtonous cultures, lactic acid bacteria, *Lactobacillus pentosus*, Tonda di Cagliari, table olive, phenolic compounds, oleuropein

## Abstract

Twenty-seven *Lactobacillus pentosus* strains, and the undefined starter for table olives from which they were isolated, were characterised for their technological properties: tolerance to low temperature, high salt concentration, alkaline pH, and olive leaf extract; acidifying ability; oleuropein degradation; hydrogen peroxide and lactic acid production. Two strains with appropriate technological properties were selected. Then, table olive fermentation in vats, with the original starter, the selected strains, and without starter (spontaneous fermentation) were compared. Starters affected some texture profile parameters. The undefined culture resulted in the most effective *Enterobacteriaceae* reduction, acidification and olive debittering, while the selected strains batch showed the lowest antioxidant activity. Our results show that the best candidate strains cannot guarantee better fermentation performance than the undefined biodiverse mix from which they originate.

## 1. Introduction

Table olives are the most widely diffused traditional fermented vegetable product in the Mediterranean area [1]. The process is performed with the purpose of reducing olives bitterness to a palatable level, to enhance sensory features, while ensuring safety of consumption via acidification and/or biopreservation [2]. Natural fermentation is carried out by soaking raw olives in brines (6%–10% NaCl), where environmental microflora colonizing olives, vats, and tools used in previous processes give rise to a spontaneous fermentation, driven mainly by lactic acid bacteria (LAB) and yeasts. To improve the onset of favourable physical–chemical conditions during the early process stages, brines from previous fermentations can be used as microbial inoculum for new batches, according to the back-slopping method [3,4]. Thus, in several productions, natural fermentation is replaced by the use of microbial starters, yeast- or LAB-based, to enhance the fermentation performances, speeding up the acidification of brines [2], preventing the proliferation of spoilage bacteria [5], or conferring probiotic characteristics to the product [6,7]. The microbial starters used for table olives can be made by few (or even one) species and strains, as in the case of the selected starter cultures, or can consist of an indefinite number of microorganisms; in this case, we refer to natural biodiverse starter cultures [2]. 

Selected starters, frequently used in industrial productions [8], control the fermentation process and standardise the end product [9] by rapid domination of the indigenous microflora of raw olives, but reduce microbial biodiversity and sensory complexity of fermented table olives [4,10]. The microbial strains forming the selected starters are chosen based on their ability to survive to brine and adverse environmental conditions, i.e., high pH and NaCl concentration, and low temperature [11], and on their ability to hydrolyse oleuropein, produce aromas, and counteract the development of spoilage microorganisms and pathogens (e.g., *Enterobacteriaceae*, *Clostridium*, *Pseudomonas*, *Staphylococcus*, and *Listeria*) [4,12,13]. On the contrary, the use of undefined biodiverse starters, composed by autochthonous microflora, better adapted to the raw olives than allochthonous ones [14], could be advantageous in terms of taste richness, linking the product with the territory of production, in case of PDO and IGP products [2]. Moreover, the undefined biodiverse starters, characterised by a large number of strains [15], are more resistant to phage attacks, which is strain-specific, and phage-insensitive strains can mutually compensate for the loss of metabolic pathways of the sensitive strains attacked [11].

Recently, Campus et al. [16] and Comunian et al. [17] reported a new technological approach using a semi-natural starter culture (SIE, selected inoculum enrichment) consisting of an undefined number of *Lactobacillus pentosus* strains obtained from a natural fermentation of table olives of the variety *Tonda di Cagliari*, a local cultivar from Sardinia, Italy [18,19]. The SIE undefined mix of autochthonous strains was more adapted to the raw olives and brine conditions than the allochthonous selected starter, showing better technological performances. Natural biodiverse starters could be advantageous over single or dual strains, since complex microbial communities have undergone natural selection, adapting to specific environmental conditions. 

In this study, 27 LAB strains were characterized for their technological properties in order to select the best candidates to be used as starters for table olives processing. The aim of this study was to compare the fermentation of table olives of the variety *Tonda di Cagliari* in brines inoculated with the autochthonous and undefined biodiverse starter (SIE), a selected double-strain starter (DSS), and natural fermentation (NF) without a starter. 

## 2. Materials and Methods

### 2.1. Experimental Plan

A biodiverse *L. pentosus* starter culture (SIE, selected inoculum enrichment) obtained from a previous successful fermentation [16] and 27 *L. pentosus* strains, previously molecularly biotyped [17], were characterised for their technological features: 11 strains were isolated from the SIE starter; 14 came from vats of table olives inoculated with SIE; 2 from vats of table olives under natural fermentation. Two strains with appropriate technological properties, belonging to the 11 SIE isolates, were selected to be used as the double-strain starter (DSS) in a new table olive experimental trial, in comparison with the original SIE starter culture and natural fermentation (NF). 

### 2.2. Technological Characterisation

Cultures kept frozen at −80 °C were reactivated by streaking on De Man, Rogosa and Sharpe (MRS) agar plates, incubated at 30 °C for 24 h, in anaerobiosis. All the phenotypic tests, described in the following paragraphs, were performed in triplicate using a standard inoculum of 1.5 × 10^5^ CFU/mL. In spectrophotometric assays (BioPhotometer plus, Eppendorf AG, Hamburg, Germany), bacterial growth was expressed as optical density at 600 nm (OD_600_), and only cultures showing an OD_600_ ≥ 0.15 were considered positive.

#### 2.2.1. Tolerance to Low Temperatures, High Saline Concentrations, and Alkaline pH 

The 27 strains and the SIE starter culture were tested for their tolerance to low temperatures in MRS broth, at 10 and 15 °C, after 3 and 7 days of incubation. 

Tolerance to high saline concentrations was assessed in MRS broth supplemented with 8 or 10% NaCl and incubated at 30 °C for 72 h. 

In order to test the tolerance to alkaline pH, the bacterial cultures were inoculated in half-strength MRS broth adjusted to pH 8 with NaOH 0.25 N (International System of units (SI)), and incubated at 30 °C for 48 h in anaerobiosis [20].

To test the tolerance of the cultures to low temperatures, high saline concentration and alkaline pH, the bacterial growth was evaluated spectrophotometrically.

#### 2.2.2. Bacterial Growth and Acidification Ability 

The cultures were inoculated in MRS broth and incubated at 30 °C for 24 h. Then, different aliquots were used for the pH measurement (pH meter pH510, Eutech Instruments, City, Country), and for the bacterial growth evaluation, both spectrophotometrically (OD_600_) and by plate count (Log CFU/mL), in MRS agar, incubated at 30 °C for 72 h in anaerobiosis.

#### 2.2.3. Tolerance to Olive Leaf Extract 

To test the tolerance to olive leaf extract (OLE), 5 μL of each overnight culture, at 1.5 × 10^5^ CFU/mL, were spotted on MRS agar plates supplemented with 10% (*w/v*) of OLE, and incubated at 30 °C for 72 h in anaerobiosis. OLE powder was obtained by dehydrating olive leaves at 105 °C for 24 h and then grinding with a homogenizer (Type-A10 Janke & Kunkel GmbH & Co. Kg Ika-Werk, Staufen, Germany). Strains developing colonies on the medium were considered tolerant of OLE. A negative control without OLE was included in the assay [21]. 

#### 2.2.4. Use of Oleuropein as Substrate

Modified MRS broth in which glucose was replaced with 1% (*w/v*) oleuropein (Applichem GmbH, Darmstadt, Germany) as the sole carbon source, was used for testing the oleuropein degradation ability of the microbial isolates and the SIE culture. The test was performed following a modified protocol of Ghabbour et al. [21], inoculating the cultures in a final volume of 100 µL in micro-plates. Degradation ability was assessed by visual examination of microbial growth after 7 days of incubation at 30 °C. Microplates wells showing cellular precipitate (pellet) at the bottom were considered positive. Standard MRS medium broth inoculated with the cultures was used as positive control.

#### 2.2.5. Hydrogen Peroxide Production

The ability to produce hydrogen peroxide was tested according to Marshall [22] modified by Berthier [23], using Peptonized agar medium (PTM) containing HRP (horseradish peroxidase) and ABTS (2,2’-azino-bis (3-ethylbenzothiazoline-6-sulphonic acid)) as chromogenic substrate. Five microliters of each culture were spotted onto the plates and then incubated at 30 °C for 48 h in anaerobiosis. At the end of the incubation, the plates were exposed to air for 120 min at 30 °C, and for an additional 180 min at room temperature. The peroxide production was highlighted by the colour change of the colonies, and the tested strains were assigned to five categories. In order to perform the statistical analysis, a number was arbitrarily assigned to each category as follows: colourless, non-producer (0); green halo, very weak producer (1); green, weak producer (2); light purple, producer (3); dark purple, strong producer (4). 

#### 2.2.6. Lactic Acid Production 

The test was performed on 11 strains, chosen among the best acidifying strains (tested in Section 2.2.2), and the SIE starter culture. Quantification of lactic acid D and L produced was carried out using the D-Lactic acid/L-Lactic acid Kit UV-method (R-Biopharm AG, Darmstadt, Germany), according to the manufacturer’s instructions. The results were expressed in g/L of total D/L-lactic acid produced.

### 2.3. Starter Culture Origin and Preparation

The SIE starter culture, D104 and D702 strains, chosen among the SIE isolates and joined in the DSS starter, were reactivated by inoculating 10 μL of the concentrated culture stored at −80 °C in MRS broth and incubating overnight at 30 °C. The cultures grown were inoculated at a 1% rate in fresh MRS broth and incubated under the same conditions as the day before. The cultures were centrifuged (Centrifuge SL40R, Thermo Fisher Scientific, Lagenselbold, Germany) at 4500 rpm at 2 °C for 15 min in 500 mL volume Bio-bottles (Thermo Fisher Scientific). After discarding the supernatant, the pellets were washed with 200 mL of saline solution (0.89% *w/v* NaCl), in order to eliminate medium residues, resuspended in cryoprotectant (gelatin 5%, Na-citrate 5%, monosodium glutamate 5%, sucrose 10%, pH 7), and kept frozen at −80 °C. Before use, the cell concentration of the SIE starter and the strains D104 and D702 were checked by plate count in order to prepare a suitable inoculum for the SIE and DSS brines. 

### 2.4. Pilot Scale Fermentation Trials

Olives from the variety *Tonda di Cagliari* were mechanically collected from an irrigated olive orchard, located in the south of Sardinia (Italy), at the green-yellow ripe stage. Defective fruits were discarded and then calibrated olives (fruit diameter between 17 and 20 mm) were carefully washed in tap water under continuous stirring, allowing the dripping of the excess water. The olives were placed in sanitised plastic vats that had a capacity of 220 L, filled up with NaCl brine (130 kg of olives and 90 L of 7% NaCl brine, kept constant manually throughout the process). An experimental design with 3 replicates and 3 repetitions per treatment was used. Vats were inoculated with DSS or SIE starter cultures, in order to reach an inoculum with a final concentration of 1.5 × 10^6^ CFU/mL in brine. Natural fermentation (NF) vats were prepared as control. Vats were transferred to an acclimatized room and kept at 25 °C throughout the experiment.

### 2.5. Physical-Chemical Analyses

Olive brines were analysed for pH and titratable acidity (expressed as grams of lactic acid per 100 mL brine) using standard laboratory methods. Volatile acidity (expressed in grams of lactic acid per 100 mL of brine) was carried out by steam distillation, as follows: 10 mL of brine was put in a 50 mL flask, adding 1 g of tartaric acid. Volatile acids were distilled under steam current using a distillation apparatus and decarbonized distilled water as steam feeding. The distillate (250 mL) was collected and titrated with NaOH 0.1 N, using phenolphthalein as the indicator. 

Sodium chloride in brines was determined according to the Mohr method: 1 mL of brine was diluted with 50 mL of distilled water, titrated with AgNO_3_ 0.1 N with K_2_CrO_4_ as the indicator. All chemicals were purchased from Sigma Aldrich (Milan, Italy). Samples were analysed after 0, 7, 15, 30, 60, 90, and 180 days.

### 2.6. Phenolic Analysis

Phenolic compounds extracts were obtained according to the IOC method for the determination of biophenols by HPLC in olive oils [24], with some minor changes. Three grams of homogenized olives were extracted twice with 15 mL of a methanol/ water (80/20 *v/v*) solution and 10 mL of hexane. Tubes were agitated for 20 min in a rotatory shaker, then the organic layer was separated with a separatory funnel. The two MeOH/H_2_O extracts were combined, filtered through a 0.45 µm PTFE syringe filter (Whatman Inc., Clinton, NJ, USA), and dried in a rotary evaporator Rotavapor^®^ R-300 (Buchi, Flawil, Switzerland) at 30 °C. The residue was dissolved in 15 mL of ethyl acetate, adding 2 g of anhydrous MgSO_4_ to remove the remaining water fraction. One millilitre of the ethyl acetate solution was gently dried under N_2_ stream, recollected with 1 mL of methanol and injected in HPLC/DAD for the analysis.

A HPLC 1100 (Agilent Technologies, Milan, Italy) equipped with a DAD detector UV 6000 (Thermo Finnigan, Milan, Italy) was used. The column was a Varian Polaris C18 (5 µm, 300 A, 250 X 4.6 mm). Analyses were carried out at 280 and 360 nm, in gradient elution. Solvents were phosphoric acid 0.22 M (A), acetonitrile (B), and methanol (C), and the gradient program (T= time, in minutes) was: T = 0 A 96%, B and C 2%; T = 40 A 50%, B and C 25%; T = 45 A 40%, B and C 30%; T = 60 A 0%, B and C 50%, hold: 10 min; post time: 15 min., flow: 1 mL/min. Calibration curves were prepared in the range 5–50 µg/mL of authentic analytical standards of tyrosol, 3-hydroxytirosol, benzoic acid, paracumaric acid, ferulic acid, quercitin, luteolin, oleuropein, verbascoside and apigenin (Sigma-Aldrich Inc., St. Louis, MO, USA), except elenolic acid, which was synthetised in the laboratory. Stock solutions of the analytes were prepared in methanol (1000 µg/mL). Intermediate stock standard solutions were prepared at 100 µg/mL in methanol by dilution of stock standard solutions. Working standard solutions were prepared in methanol and used for qualitative and quantitative analysis.

### 2.7. DPPH Scavenging Activity as Trolox Equivalent Antioxidant Capacity (TEAC)

Five grams of destoned olives were homogenized, added with 10 mL of methanol and vigorously stirred for 20 minutes, then centrifuged at 4000 rpm for 25 min. DPPH-free radical scavenging capacity of phenolic extracts was evaluated according to the following protocol: 200 μL of the extracts or standard (Trolox) was added to 3 mL methanol solution of DPPH radical. After 1 min of vigorous shaking by vortex, the reaction mixture was left to stand at room temperature, in the dark, for 60 min. After that, the absorbance for the sample was read using a Varian Cary 50 UV–vis spectrophotometer (Varian Inc., Middelburg, The Netherlands), at λ = 517 nm, optical path 10 mm. A negative control was taken after adding the DPPH solution to the respective extraction solvent. The free radical scavenging capacity was expressed in Trolox equivalents (TE), e.g., mmol TE/kg, and quantified against a calibration curve of Trolox (*r* = 0.99). 

### 2.8. Texture Analyses

Texture profile analyses (TPA) were carried out with a TA-XT Plus texture analyser (Stable Microsystems, Surrey, UK) with a plugged 30 kg load cell, coupled with the Exponent software (ver. 6.1.3.0) for acquisition and processing. Analyses were carried out on 30 fruits for each replicate, for a total of 90 fruits for each experimental condition. Olives were put on the heavy-duty platform and compressed along the longitudinal side by 15% of their thickness with the P/40 aluminium cylinder. Test speed was set at 1 mm/sec, time between compressions was 2 sec, and trigger force was set at 0.05 N. The TPA parameters computed were hardness, cohesiveness, gumminess, chewiness and springiness, according to Szczesniak [25] and Friedman et al. [26].

### 2.9. Microbiological Analyses

Samples of uninoculated brines, used for all the experimental theses, were collected. Decimal serial dilutions in saline solution (0.89% *w/v* NaCl) were prepared and plated, in duplicate, on FH agar medium, incubated at 30 °C for 72 h in anaerobiosis, for mesophilic lactobacilli enumeration; MEA agar medium (Microbiol, Uta Cagliari) supplemented with 0.01% of chloramphenicol (Sigma-Aldrich), incubated at 25 °C in aerobiosis, for yeasts and moulds; VRBGA medium (Microbiol), incubated at 30 °C for 18–24 h in aerobiosis, for *Enterobacteriaceae*. Furthermore, olives before brining and olives after 7, 15, 30, 60, 90, and 180 days from brining were collected. Samples constituting 130 g of olives and 90 mL of saline solution for olives before brining, or fermentation brine, were collected and homogenized for 10 min by a BagMixer paddle blender (Interscience Corporation, Saint Nom, France). Microbial counts were performed in duplicate on the growth media and incubation conditions indicated above. Analyses were performed on three vats for each experimental thesis (SIE, DSS and NF) and expressed as average Log CFU/mL.

### 2.10. Statistical Analysis

One-way analysis of variance (ANOVA) for the evaluation of significance (*P* < 0.05) was performed on the whole data set. Differences between the individual means were compared by Tukey’s HSD post hoc test, using the software SPSS Statistics (v. 21.0; IBM Corp., Armonk, NY, USA).

## 3. Results

### 3.1. Technological Characterisation 

The technological characterisation was based on the tolerance to low temperature, high saline concentration and alkaline pH, OLE resistance, oleuropein degradation, and acidification ability. Moreover, hydrogen peroxide and lactic acid production were also investigated.

#### 3.1.1. Tolerance to Low Temperatures, High Saline Concentrations, and Alkaline pH

None of the isolates or the SIE starter culture were able to grow at 10 °C (data not shown). The bacterial growth was observed only at 15 °C after 7 days of incubation, and no significant (*P* < 0.05) differences were generally observed among the strains and the SIE culture, with few exceptions (Table 1). Most of the cultures tolerated saline concentrations up to 8% NaCl (*w/v*). D102, D104, D702 and SBOD300 strains showed better adaptability to the brine conditions. Only D714, D723, FNI901, SBOF1002, and SBOF901 strains were not able to grow (Table 1). None of the isolates and the SIE starter culture tolerated 10% NaCl.

All the cultures were able to grow in alkaline MRS (pH 8) after 48 h, and significant (*P* < 0.05) differences were observed among a few of the isolated tested. In particular, the growth of D713 was significantly lower than that of SBOE1000 and SBOE802, whereas SBOF901 showed a significantly lower growth than SBOE802 (Table 1).

#### 3.1.2. Bacterial Growth and Acidification Ability 

The growth of the isolates and the SIE culture was tested at 30 °C, and it was measured both optically (OD_600_) and by plate count agar (CFU/mL). The OD_600_ values ranged from 3.80 of D723 to 7.60 of D724, whereas the number of CFU/mL ranged from 7.73 of D723 to 9.07 of D104 (Table 1). No significant (*p* < 0.05) differences in microbial growth after 24 h of incubation were observed among the cultures using both detection methods.

The acidification performance after 24 h was also evaluated. The final pH ranged between 4.07 of D710 and 4.68 of D723, and, similarly to as observed for the bacterial growth, no significant (*P* < 0.05) differences among the cultures were calculated (Table 1).

#### 3.1.3. Olive Leaf Extract Tolerance and Use of Oleuropein as Substrate

All the isolates and the SIE culture were tolerant to 10% of OLE and showed degradation of 1% oleuropein.

#### 3.1.4. Hydrogen Peroxide Production

**Figure 1:**
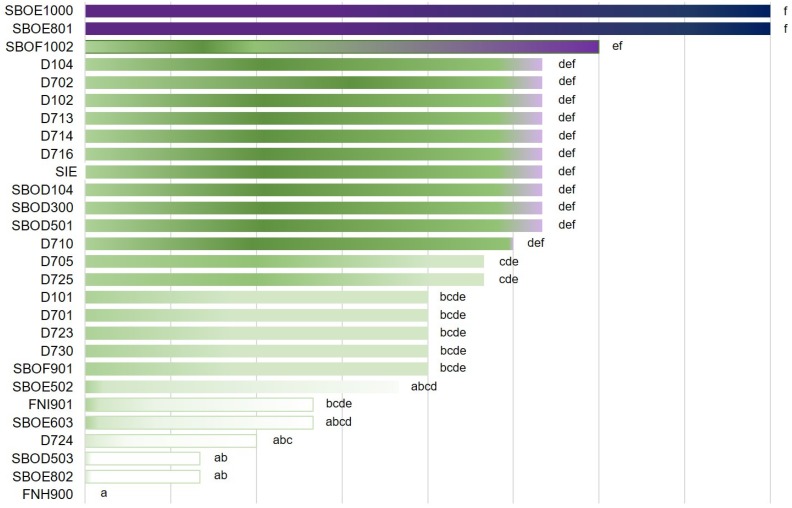
Hydrogen peroxide production of the characterised isolates and the semi-natural starter culture (SIE) starter culture. For each microbial culture tested, rows sharing the same letters do not differ significantly (*P* < 0.05), according to Tukey’s HSD post hoc test.

The isolates revealed different levels of hydrogen peroxide production, with significant (*P* < 0.05) differences among the cultures. SBOE1000 and SBOE801 showed the highest production, which was not significantly (*P* < 0.05) higher than the isolates D104 and D702 (subsequently joined in the DSS culture), and the SIE culture (Figure 1). 

#### 3.1.5. Lactic Acid Production

The production of D, L, and total lactic acid revealed an interesting scenario among the bacterial cultures characterised. Significant (*P* < 0.05) differences in the amount of lactic acid produced were observed among the isolates, and generally, the SIE culture produced less lactic acid than most of the isolates (Table 2).

Based on the results obtained by the technological characterisation, two strains (D104 and D702) from the SIE undefined culture were selected and joined to make the double-strain starter (DSS) for table olive fermentation in vats. These two strains were among the best hydrogen peroxide producers and tolerated low temperature (i.e., 15 °C), high saline concentration (NaCl 8%), alkaline pH (8), and OLE (10%). Furthermore, their capacity to grow at the temperatures tested in this work (15 and 30 °C), the acidification ability, and the lactic acid production were comparable and not significantly different to the SIE culture. 

### 3.2. Microbiological Analyses

Preliminary investigation on uninoculated brines and olives before brining revealed a very low yeast contamination (1.82 and 3.49 Log CFU/mL, respectively), while mesophilic lactobacilli were not detected. *Enterobacteriaceae* were found only in the olives (4.60 Log CFU/mL).

**Figure 2:**
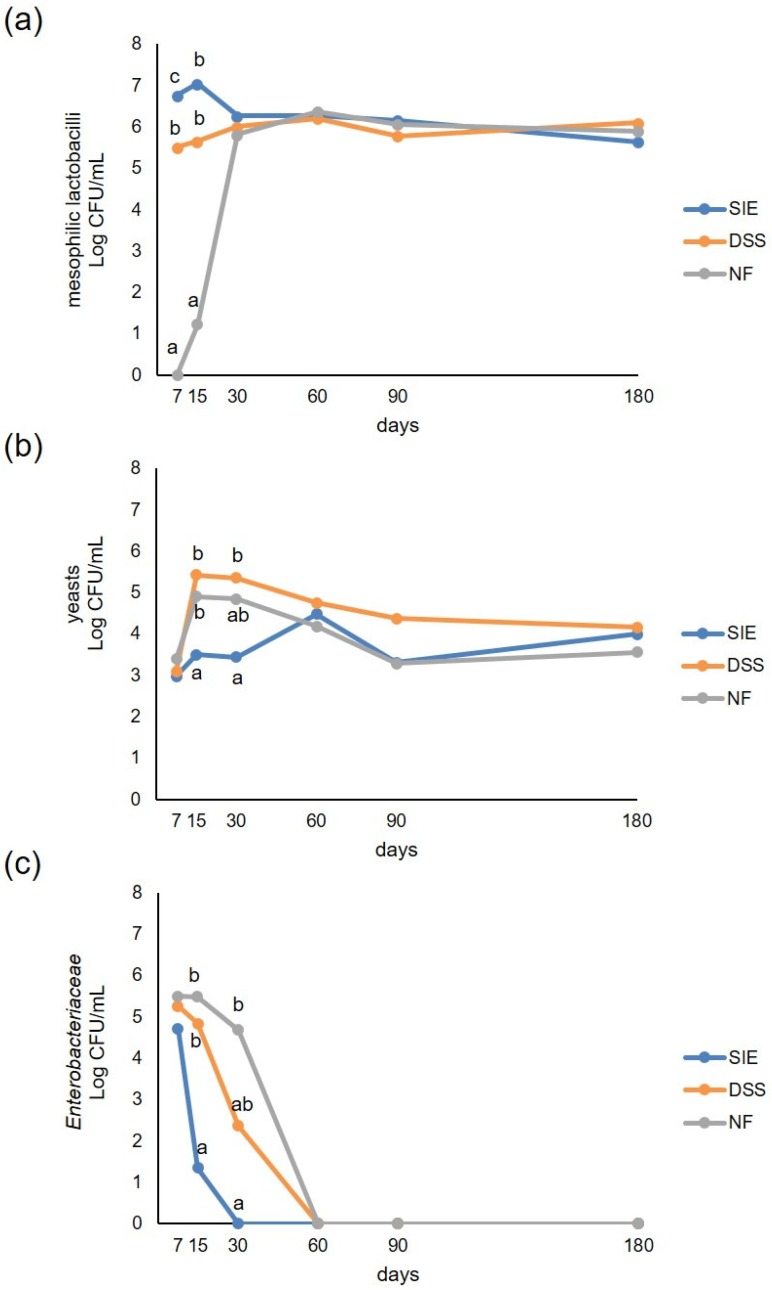
Microbial counts of viable mesophilic lactobacilli (**a**), yeasts (**b**), and *Enterobacteriaceae* (**c**) in vats inoculated with SIE and double-strain starter (DSS) starter cultures, and with natural fermentation (NF), evaluated after 7, 15, 30, 60, 90, and 180 days from the inoculum. For each microbial group and time-point of detection, counts, expressed as Log CFU/mL, sharing the same letters do not differ significantly (*P* < 0.05), according to Tukey’s HSD post hoc test.

After 7 days from the inoculum in brine, mesophilic lactobacilli were below the level of detectability in NF samples, while reached 6.76 and 5.51 Log CFU/mL in SIE and DSS, respectively (Figure 2a). During the early stage of fermentation, higher counts were found in SIE than in DSS, showing better adaptability of the undefined starter SIE to brine conditions. Statistical differences (*P* < 0.05) among the three theses were found up to 15 days from brining. After 30 days from the inoculum, mesophilic lactobacilli counts were comparable in the three vats, remaining constant at around 6 Log until the end of the trial.

Yeast development was well controlled by the SIE starter culture (Figure 2b), as well as the *Enterobacteriaceae* (Figure 2c). In particular, yeasts, starting from about 3 Log CFU/mL in the three theses, slightly increased throughout the incubation period in SIE, whereas they were about 2 Log higher (*P* < 0.05) in DSS and NF at 15 and 30 days. At 60 days, yeasts reached similar levels in all the theses, then tended to decrease reaching a concentration between 3.56 Log CFU/mL (SIE) and 4.15 Log CFU/mL (DSS) at 180 days.

*Enterobacteriaceae* were about 5 Log CFU/mL after 7 days from brining in all the three theses. During the first 30 days of incubation, they decreased rapidly, not being detectable in SIE samples, while in NF and DSS *Enterobacteriaceae* were no more detectable from the 60th day.

Moulds were never found in all of the samples analysed.

### 3.3. Physical-Chemical Analyses

**Figure 3:**
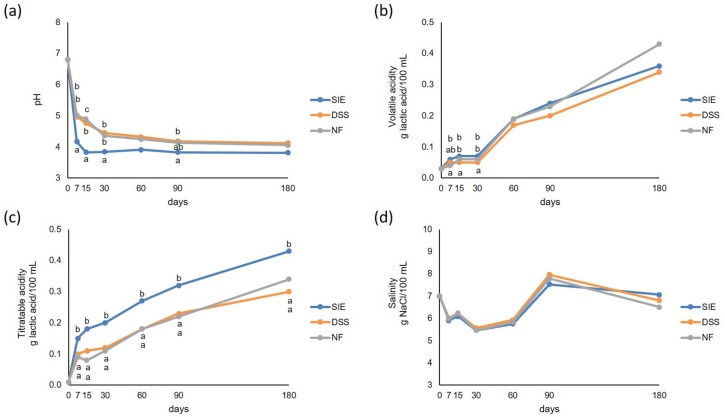
Physical–chemical parameters evolution during fermentation. (**a**) pH, (**b**) volatile acidity (g of lactic acid/100 mL), (**c**) titratable acidity (g of lactic acid/100 mL), and (**d**) salinity (*w/v*), measured immediately after the inoculum (0 d) and after 7, 15, 30, 60, 90, and 180 days. For each parameter and sampling time, values sharing the same superscript letters (if present) do not differ significantly (*P* < 0.05), according to Tukey’s HSD post hoc test.

No differences were observed in salinity among the three theses throughout the fermentation (Figure 3). Generally, DSS and NF showed not significant (*P* < 0.05) differences in titratable acidity and pH values. On the contrary, SIE showed significantly (*P* < 0.05) higher values during the evolution of titratable acidity. The monitoring of volatile acidity revealed significant differences between DSS and the other theses, which showed slightly higher values throughout the trial. A rapid fall in pH was observed in SIE, reaching values lower than 4 in 15 days, remaining almost constant until the end of observations (at 180 days, pH was 3.81), while DSS and NF never reached pH < 4 till the end of the trial (4.12 and 4.06, respectively). 

### 3.4. Phenolic Compounds Concentration and Antioxidant Activity as TEAC (Trolox Equivalent Antioxidant Capacity)

The HPLC analysis of phenols in the pulp in the different treatments showed 13 main compounds accounting for almost 90% of total phenols detected. For most of the individual phenols, significant (*P* < 0.05) differences between the concentrations were detected. Hydroxytyrosol was the most abundant in all samples, showing higher levels in SIE, according to the negligible values of oleuropein in these samples (Table 3), followed by verbascoside. Elenoic acid, 4-OH benzoic acid, paracumaric acid, quercetin dihydrate, and apigenin showed similar values in all treatments, while tyrosol, luteolin, luteolin 7-glucoside, and the unknown compound showed higher values in SIE samples, and comparable amounts in DSS and NF treatments.

Oleuropein was not detectable in SIE samples while showed comparable amounts in DSS and NF in vitro antioxidant activity as TEAC was comparable among the theses although DSS showed the lowest values.

### 3.5. Texture Analyses 

The TPA tests, carried out at the end of the fermentation, showed no differences (*P* < 0.05) among olives from the three theses in all texture parameters except for “gumminess” and “chewiness” (Table 4). “Gumminess” is “hardness × cohesiveness”, thus this parameter refers to the “solidity” of the material and its resistance to deformation. “Chewiness” is “gumminess × elasticity”. SIE samples showed significantly higher values of these parameters. 

## 4. Discussion

To answer the question raised in the title, two strains (D104 and D702), chosen among the best performers, isolated from the autochthonous SIE starter, were used as a double-strain starter (DSS) in a table olive fermentation trial, in comparison with the SIE starter culture and natural fermentation (NF). Overall, the SIE starter carried out the fermentation with better results than DSS and NF, even though D104 and D702 showed better performances in the technological characterisation tests. These strains showed among the best peroxide production and resistance to salt (i.e., 8% NaCl) performances, while the oleuropein hydrolysis and growth after 24 h, at all the temperatures tested, were comparable to the SIE culture, as well as the acidification ability. During the fermentation, SIE pushed more acidification, lowering the pH to a value <4.0, which is fundamental for the preservation of table olives since it prevents the proliferation of harmful and spoilage bacteria [5]. The pH drop observed during fermentation is due to the conversion of carbohydrates into organic acids, mainly lactic acid, by LAB fermentation. In addition, the hydrolysis of oleuropein, which is decomposed by endogenous and bacterial enzymes in sugars and simple phenols such as OH tyrosol and elenolic acid, may contribute to the pH fall and acidity rise [27]. The use of the starters (SIE and DSS) revealed a greater performance in controlling the evolution of spoilage bacteria and the development of favourable physical–chemical conditions during the fermentation compared to NF. The effectiveness of the starter culture addition was also observed in yeast control and the *Enterobacteriaceae* reduction, greater in the SIE vats, where, in the early fermentation phase, mesophilic lactobacilli were almost 1 and 6 Log CFU/mL higher than in DSS and NF, respectively. Interestingly, in NF, despite mesophilic lactobacilli slowly developed and reached the same level found in SIE and DSS only after 30 days of fermentation, it was observed that there was a pH trend similar to DSS, since a contribution to pH decrease could also come from the diffusion of organic acids from pulp to brine. The pH decreasing is involved in the prevention of spoilage microorganisms and pathogen contamination requested for table olive safety [28]. Indeed, *Enterobacteriaceae*, which could cause infections in humans and be responsible for table olive defects such as gas pockets formation, are the first microbial group able to grow during the early olive fermentation but are rapidly supplanted by LAB [29] through the decrease of pH [30]. Therefore, the use of the SIE starter could be a good hygiene practice in table olive processing, according to Campus et al. [16,31]. The faster disappearance of *Enterobacteriaceae*, as observed in the SIE thesis, has beneficial effects also on the table olive sensory quality [32]. 

Yeasts are involved in milder taste defects, excessive CO_2_ production, and olive cells wall degradation [33,34]. However, yeasts can even improve the final product by the production of volatile compounds and the enhancement of LAB growth [34,35,36]. In this study, the vats inoculated with the SIE starter culture showed an almost constant yeast concentration throughout the fermentation, lower than in NF and in vats inoculated with the DSS starter. Due to its biodiversity, the SIE culture could have limited and better regulated yeast development during the fermentation. 

The main phenomena responsible for changes in phenolic concentrations are the osmotic dehydration and the enzymatic activity exerted by endogenous and microbial enzymes. Olives are submerged in a hypertonic medium (brine), and plant tissues act as semipermeable membranes in relation to water movements when immersed in a hypertonic solution [37]. During the process, two major countercurrent flows take place simultaneously. The setting up of gradients across the product–medium interface leads to water flows from the product into the osmotic solution, whereas osmotic solute (NaCl) is transferred from the solution into the product. As a result, table olives increase in salt content during processing and lose sugars, phenols, acids, minerals, and vitamins into the solution [38]. The rate of diffusion varies according to the concentration and temperature of the osmotic solution, size and geometry of the material, solution to material mass ratio, and level of agitation of the solution [39]. The lower content of oleuropein in SIE is due to enzymatic hydrolysis carried out by inoculated lactic acid bacteria with β-glycosidase and esterase activity. Hydroxytyrosol, together with elenolic acid, derives from the hydrolysis of oleuropein by β-glycosidases and esterases, enzymes of endogenous and microbial origin. As reported by Cardoso et al. [40], hydroxytyrosol was the most abundant phenolic compound in MeOH extracts of olive pulp. Marsilio et al. [41] reported that in processed Greek-style table olives coming from var. *Ascolana tenera*, both naturally fermented and inoculated with a *Lactobacillus plantarum* based starter culture, oleuropein and hydroxytyrosol were the most abundant phenols.

The olives analysed in this study resulted overall in comparable texture, although the SIE samples showed a significantly higher resistance to deformation, as shown by the gumminess and chewiness parameter magnitudes. As reported in literature [42], changes in texture during natural fermentation of olives can be ascribed to hydrolysis of cell wall pectic polysaccharides, which results in loss of structural coherence of olive tissues, as observed by Servili et al. [43] with SEM techniques.

Recently, Bleve et al. [13] described a selection procedure for the production of mixed autochthonous starters for table olive fermentation. The autochthonous starters, isolated from the microbiota of raw olives, could have the advantage of being better adapted to the matrix to be processed than the allochthonous ones, with extended shelf-life [44] and better sensory quality of the final product [3,14,45]. Moreover, the use of biodiverse and complex microbial communities as starter cultures, instead of the mono- or two-selected strains frequently employed [11], is advantageous in terms of resistance against phage attacks and possible failure of the fermentation [5]. Phage infections are usually strain-specific and, in case of attack, in a biodiverse culture, the other phage-insensitive strains can survive and compensate for the lack of the sensitive-strains [2]. 

## 5. Conclusions

In this study, the SIE starter, an undefined mix of autochthonous *L. pentosus* strains, has been shown to be more efficient in brine acidification, leading to a safer product, supplanting spoilage bacteria earlier than the DSS starter and natural fermentation. Debittering was achieved in a shorter time. The hydrolysis of oleuropein into elenolic acid and hydroxytyrosol was more intense using the SIE starter, resulting in a higher amount of most of the phenolic compounds compared to the double-strain starter. Moreover, instrumental texture was not substantially affected by the use of microbial starters. Overall, the DSS did not reach the same performances of the SIE starter, showing behaviour similar to NF or in-between the two experimental theses. 

Autochthonous complex microbial communities coming from the same environment of the raw material to be processed have more adaptability to harsh fermentation conditions, preserving safety and quality characteristics of naturally fermented olives faster, thus reducing production costs. 

## Figures and Tables

**Table 1:** Technological properties (growth at low temperature, high salinity and alkaline pH) of isolates and natural communities.

Culture	Growth at 15 °C 7 day	Growth NaCl 8% 3 day	Growth pH 8 48 h	Growth 30 °C 24 h		pH 24 h
	OD600	OD600	OD600	OD600	CFU/mL	UpH
D101	1.00 ± 1.86 ^abc^	1.07 ± 0.90	4.56 ± 0.36 ^abc^	6.09 ± 0.31	8.96 ± 0.28	4.29 ± 0.09
D102	1.05 ± 1.20 ^abc^	2.04 ± 0.65	4.13 ± 0.23 ^abc^	6.14 ± 0.59	8.66 ± 0.49	4.15 ± 0.02
D104	0.26 ± 0.33 ^a^	2.11 ± 0.27	4.33 ± 0.11 ^abc^	6.28 ± 0.82	9.07 ± 0.35	4.16 ± 0.05
D701	3.56 ± 1.90 ^abc^	0.50 ± 0.87	4.07 ± 0.42 ^abc^	6.12 ± 1.00	8.41 ± 0.33	4.18 ± 0.07
D702	0.36 ± 0.34 ^a^	2.01 ± 0.49	4.30 ± 0.10 ^abc^	6.27 ± 0.45	8.58 ± 0.50	4.15 ± 0.03
D705	4.25 ± 0.33 ^abc^	0.99 ± 1.15	4.38 ± 0.05 ^abc^	6.49 ± 0.78	8.81 ± 0.50	4.05 ± 0.03
D710	4.01 ± 0.77 ^abc^	1.02 ± 1.43	3.95 ± 0.29 ^abc^	6.51 ± 1.07	8.17 ± 0.85	4.07 ± 0.04
D713	3.38 ± 2.71 ^abc^	0.80 ± 1.39	3.66 ± 0.32 ^a^	6.58 ± 2.13	8.74 ± 0.37	4.17 ± 0.19
D714	4.02 ± 1.95 ^abc^	0.00 ± 0.00	3.95 ± 0.32 ^abc^	5.68 ± 2.61	8.42 ± 0.67	4.34 ± 0.33
D716	4.33 ± 1.67 ^abc^	0.44 ± 0.75	4.20 ± 0.39 ^abc^	6.12 ± 1.11	8.28 ± 0.52	4.21 ± 0.13
D723	4.67 ± 1.62 ^abc^	0.00 ± 0.00	4.00 ± 0.64 ^abc^	3.80 ± 2.89	7.73 ± 0.79	4.68 ± 0.59
D724	4.62 ± 0.67 ^abc^	0.57 ± 0.98	4.48 ± 0.27 ^abc^	7.60 ± 0.55	8.34 ± 0.56	4.13 ± 0.05
D725	4.12 ± 1.47 ^abc^	0.43 ± 0.74	3.97 ± 0.64 ^abc^	6.47 ± 2.27	8.33 ± 0.49	4.15 ± 0.11
D730	3.43 ± 1.32 ^abc^	0.26 ± 0.45	4.34 ± 0.10 ^abc^	6.24 ± 1.26	8.53 ± 0.57	4.15 ± 0.17
SIE	3.74 ± 2.16 ^abc^	1.09 ± 0.78	4.45 ± 0.33 ^abc^	5.26 ± 0.82	8.19 ± 0.78	4.42 ± 0.16
FNH900	2.80 ± 2.29 ^abc^	0.67 ± 0.58	4.58 ± 0.18 ^abc^	5.12 ± 1.70	8.46 ± 0.24	4.54 ± 0.36
FNI901	0.44 ± 0.78 ^ab^	0.00 ± 0.00	4.01 ± 0.55 ^abc^	7.16 ± 0.43	8.05 ± 0.14	4.12 ± 0.02
SBOD104	0.98 ± 0.91 ^abc^	1.91 ± 0.58	4.45 ± 0.11 ^abc^	5.78 ± 0.82	8.65 ± 0.50	4.32 ± 0.06
SBOD300	1.09 ± 1.12 ^abc^	2.19 ± 0.29	4.47 ± 0.09 ^abc^	6.22 ± 0.61	8.91 ± 0.58	4.21 ± 0.08
SBOD501	3.01 ± 2.18 ^abc^	1.85 ± 0.45	4.04 ± 0.44 ^abc^	5.70 ± 1.68	8.30 ± 0.31	4.29 ± 0.03
SBOD503	5.09 ± 0.35 ^bc^	0.89 ± 1.14	4.00 ± 0.90 ^abc^	4.85 ± 3.23	8.57 ± 0.98	4.59 ± 0.74
SBOE1000	4.60 ± 0.30 ^abc^	0.35 ± 0.61	4.85 ± 0.15 ^bc^	6.89 ± 1.12	8.36 ± 0.55	4.15 ± 0.30
SBOE502	5.35 ± 0.92 ^c^	1.26 ± 0.79	4.57 ± 0.30 ^abc^	6.66 ± 1.81	7.98 ± 0.21	4.23 ± 0.08
SBOE603	0.81 ± 1.53 ^abc^	1.00 ± 1.31	4.11 ± 0.46 ^abc^	6.19 ± 1.09	8.43 ± 0.45	4.14 ± 0.11
SBOE801	2.42 ± 1.90 ^abc^	0.21 ± 0.32	4.78 ± 0.16 ^abc^	4.50 ± 3.00	7.99 ± 1.53	4.30 ± 0.14
SBOE802	3.86 ± 2.68 ^abc^	0.35 ± 0.15	5.05 ± 0.25 ^c^	6.06 ± 1.25	8.32 ± 0.58	4.31 ± 0.05
SBOF1002	1.99 ± 2.10 ^abc^	0.00 ± 0.00	4.59 ± 0.45 ^abc^	4.15 ± 3.46	8.40 ± 0.64	4.21 ± 0.09
SBOF901	3.49 ± 1.74 ^abc^	0.00 ± 0.00	3.86 ± 0.32 ^ab^	4.38 ± 3.29	8.60 ± 0.26	4.26 ± 0.11

Technological test performed for microbial isolates and natural communities. Adsorbance at 600 nm (OD_600_), enumeration of CFU/mL, and pH measuring were evaluated after 24 h, 48 h, 3 days, or 7 days (mean values ± SD, *n* = 3). For each parameter, average values sharing the same superscript letters (if present) do not differ significantly (*P* < 0.05), according to Tukey’s HSD post hoc test.

**Table 2:** Lactic acid production of selected bacterial isolates.

Culture	Lactic acid D− (g/L)	Lactic acid L+ (g/L)	Total Lactic acid (g/L)
D101	6.41 ± 0.82 ^abcd^	2.53 ± 0.41 ^abc^	9.55 ± 0.93 ^ab^
D102	7.30 ± 0.97 ^bcde^	3.09 ± 0.95 ^abc^	10.40 ± 0.61 ^ab^
D104	5.69 ± 0.39 ^abc^	2.54 ± 0.25 ^abc^	8.21 ± 0.45 ^ab^
D702	7.94 ± 1.04 ^cde^	2.82 ± 0.92 ^abc^	11.08 ± 0.01 ^cde^
D705	4.21 ± 0.02 ^a^	3.43 ± 0.57 ^abc^	7.54 ± 0.75 ^cdef^
D710	7.03 ± 0.79 ^abcde^	4.03 ± 0.94 ^abc^	11.27 ± 0.44 ^def^
D724	4.81 ± 1.04 ^ab^	4.76 ± 0.21 ^c^	11.19 ± 1.09 ^def^
D730	7.08 ± 1.00 ^bcde^	4.31 ± 0.48 ^bc^	11.39 ± 0.52 ^def^
SIE	4.66 ± 0.48 ^ab^	2.18 ± 0.94 ^ab^	8.48 ± 0.90 ^ab^
FNI901	8.90 ± 0.95 ^def^	1.94 ± 0.56 ^a^	10.12 ± 0.15 ^def^
SBOE1000	11.53 ± 0.23 ^f^	2.18 ± 0.95 ^ab^	12.71 ± 0.85 ^ef^
SBOE603	9.46 ± 0.35e ^f^	2.36 ± 0.44 ^ab^	11.82 ± 0.74 ^f^

Concentration (mean values ± SD, *n* = 3) of lactic acid D−, L+, and DL produced by selected bacterial isolates and natural communities. For each isomeric form of lactic acid, average values sharing the same superscript letters do not differ significantly (*P* < 0.05), according to Tukey’s HSD post hoc test.

**Table 3:** Phenolic compounds concentration (mg/kg ± SD) and TEAC activity.

Phenolic Compounds	SIE	DSS	NF
Elenolic acid	44.44 ± 8.46 ^a^	31.76 ± 4.24 ^a^	41.64 ± 7.80 ^a^
OH tyrosol	264.22 ± 5.20 ^b^	214.51 ± 9.87 ^a^	217.08 ± 27.75 ^a^
Tyrosol	34.74 ± 2.08 ^b^	25.25 ± 1.99 ^a^	25.27 ± 2.78 ^a^
4 OH benzoic acid	21.46 ± 1.82 ^a^	16.68 ± 2.64 ^a^	19.79 ± 6.23 ^a^
unknown	8.87 ± 0.47 ^b^	5.12 ± 0.51 ^a^	5.54 ± 1.04 ^a^
Paracumaric acid	9.59 ± 1.29 ^a^	11.69 ± 2.07 ^a^	18.85 ± 3.35 ^b^
Ferulic acid	6.11 ± 1.01 ^ab^	4.97 ± 0.11 ^b^	7.82 ± 1.09 ^a^
Verbascoside	175.14 ± 16.57 ^b^	124.57 ± 6.09 ^a^	130.96 ± 19.31 ^a^
Luteolin 7-glucoside	9.38 ± 2.21	n.d.	n.d.
Oleuropein	n.d.	17.05 ± 1.75 ^a^	21.01 ± 3.64 ^a^
Quercetin dihydrate	1.13 ± 0.28 ^a^	2.41 ± 0.45 ^a^	3.10 ± 0.55 ^a^
Luteolin	30.50 ± 3.52 ^b^	15.17 ± 1.25 ^a^	15.59 ± 3.11 ^a^
Apigenin	2.24 ± 0.23 ^a^	1.98 ± 0.34 ^a^	1.98 ± 0.41 ^a^
Total phenolic compounds	3942.93 ± 478.78 ^a^	3977.64 ± 612.15 ^a^	4182.20 ± 213.90 ^a^
TEAC	350.36 ± 33.82 ^a^	339.95 ± 43.38 ^a^	350.55 ± 63.12 ^a^

Concentration of main phenolic compounds identified in pulp extracts and antioxidant activity as TEAC (Trolox equivalent antioxidant capacity). For each compound, average values (n = 3) sharing the same superscript letters do not differ significantly (*P* < 0.05) according to Tukey’s HSD post hoc test. n.d.: not detected.

**Table 4:** Texture evaluation in olives at the end of fermentation.

TPA Parameters	SIE	DSS	NF
Hardness (g)	2397.31 ± 506.84 ^a^	2185.96 ± 560.90 ^a^	2209.41 ± 530.11 ^a^
Springiness	0.64 ± 0.05 ^a^	0.62 ± 0.06 ^a^	0.62 ± 0.06 ^a^
Cohesiveness	0.52 ± 0.05 ^a^	0.50 ± 0.04 ^a^	0.51 ± 0.05 ^a^
Gumminess	1228.17 ± 236.15 ^b^	1084.29 ± 251.77 ^a^	1117.14 ± 239.66 ^a^
Chewiness (g/mm)	782.25 ± 157.29 ^b^	673.48 ± 168.16 ^a^	690.67 ± 154.63 ^a^
Resilience	0.27 ± 0.03 ^a^	0.26 ± 0.03 ^a^	0.26 ± 0.03 ^a^

For each TPA parameters, average values (± SD, *n* = 3) sharing the same superscript letters do not differ significantly (*P* < 0.05), according to Tukey’s HSD post hoc test.
